# Isolating the white matter circuitry of the dorsal language stream: Connectome‐Symptom Mapping in stroke induced aphasia

**DOI:** 10.1002/hbm.25647

**Published:** 2021-09-01

**Authors:** Vatche Baboyan, Alexandra Basilakos, Grigori Yourganov, Chris Rorden, Leonardo Bonilha, Julius Fridriksson, Gregory Hickok

**Affiliations:** ^1^ Department of Cognitive Science University of California Irvine California USA; ^2^ Department of Communication Sciences and Disorders University of South Carolina Columbia South Carolina USA; ^3^ Department of Psychology University of South Carolina Columbia South Carolina USA; ^4^ Department of Neurology Medical University of South Carolina Columbia South Carolina USA

**Keywords:** aphasia, arcuate fasciculus, area spt, connectomics, conduction aphasia, dorsal premotor cortex, partial least squares, speech repetition, superior longitudinal fasciculus

## Abstract

The application of ℓ1‐regularized machine learning models to high‐dimensional connectomes offers a promising methodology to assess clinical‐anatomical correlations in humans. Here, we integrate the connectome‐based lesion‐symptom mapping framework with sparse partial least squares regression (sPLS‐R) to isolate elements of the connectome associated with speech repetition deficits. By mapping over 2,500 connections of the structural connectome in a cohort of 71 stroke‐induced cases of aphasia presenting with varying left‐hemisphere lesions and repetition impairment, sPLS‐R was trained on 50 subjects to algorithmically identify connectomic features on the basis of their predictive value. The highest ranking features were subsequently used to generate a parsimonious predictive model for speech repetition whose predictions were evaluated on a held‐out set of 21 subjects. A set of 10 short‐ and long‐range parieto‐temporal connections were identified, collectively delineating the broader circuitry of the dorsal white matter network of the language system. The strongest contributing feature was a short‐range connection in the supramarginal gyrus, approximating the cortical localization of area Spt, with parallel long‐range pathways interconnecting posterior nodes in supramarginal and superior temporal cortex with anterior nodes in both ventral and—notably—in dorsal premotor cortex, respectively. The collective disruption of these pathways indexed repetition performance in the held‐out set of participants, suggesting that these impairments might be characterized as a parietotemporal disconnection syndrome impacting cortical area Spt and its associated white matter circuits of the frontal lobe as opposed to being purely a disconnection of the arcuate fasciculus.

## Graphical abstract

The application of ℓ1‐regularized machine learning models to high‐dimensional connectomes offers a promising methodology to assess clinical‐anatomical correlations in humans. Here, we integrate the connectome‐based lesion‐symptom mapping framework with sparse partial least squares regression (sPLS‐R) to isolate elements of the connectome associated with speech repetition deficits and effectively localize the white matter circuitry of the dorsal language stream.

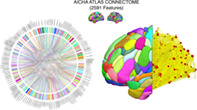

## 1 INTRODUCTION

Fluent production of speech relies heavily on auditory feedback, even for higher‐level phonological planning (Guenther & Hickok, 2015; Hickok, 2012; Hickok, 2017). A cortical circuit supporting auditory‐motor integration for speech production was discovered more than a decade ago, which includes regions in the superior temporal gyrus/sulcus, the posterior planum temporale (area Spt), the pars opercularis in Broca's region, and dorsal premotor cortex (Buchsbaum, Hickok, & Humphries, 2001; Hickok, Buchsbaum, Humphries, & Muftuler, 2003; Hickok, Okada, & Serences, 2009), which forms the dorsal speech stream (Hickok & Poeppel, 2000, 2004, 2007). However, the connectional neuroanatomy of the underlying white matter circuit of this dorsal stream network remains poorly understood (Bernal & Ardila, 2009), particularly as it relates to core clinical symptoms associated with its function.

The pioneering work of 19th and 20th century aphasiologists have shown us that cerebrovascular lesions may impair this auditory‐motor circuit leading to the syndrome of conduction aphasia (Benson et al., 1973; Geschwind, 1965; Wernicke, 1874/1977). The symptom complex of conduction aphasia includes frequent phonological errors during production and difficulty with verbatim repetition, while preserving the comprehension of speech itself (Ardila, 2010; Buchsbaum et al., 2011; Catani & Mesulam, 2008; Goodglass, 1992). Classically, the critical lesion causing conduction aphasia was thought to be the arcuate fasciculus (Geschwind, 1965), however, more recent work has implicated cortical disruption in the posterior Sylvian region as a major source of the deficits (Anderson et al., 1999; Buchsbaum et al., 2011; Damasio & Damasio, 1980; Quigg & Fountain, 1999). At the same time, it is undeniable that fluent speech production relies, at least in some part, on the integrity of subjacent white matter connections (Benson et al., 1973; Damasio & Damasio, 1980). Cerebrovascular lesions that produce conduction aphasia nearly always extend into white matter. More recently, intraoperative direct electrical stimulation studies have shown that transient phonological paraphasias can occur upon stimulation of perisylvian white matter (Duffau, 2015; Moritz‐Gasser & Duffau, 2013 for a review). Therefore, white matter involvement appears likely and mapping their organization will have important implications for resections involving white matter beneath eloquent cortical areas, which may induce undesirable speech deficits postoperatively (Ellmore, Beauchamp, O'Neill, Dreyer, & Tandon, 2009).

Although tractography studies have awarded unprecedented access to in vivo connectional neuroanatomy, the methodologies being used to make brain‐behavior correlations have lagged behind considerably. Preoccupied with validating classical assumptions, previous studies have investigated the white matter correlates of speech repetition, a core ability supported by the dorsal stream network, by restricting analyses solely to the arcuate fasciculus (Berthier, Ralph, Pujol, & Green, 2012; Forkel et al., 2020; Sierpowska et al., 2017; Yeatman et al., 2011), resulting in a spatial bias which underestimates the complexity of the underlying anatomy. Indeed, the white matter configuration of the inferior parietal lobule alone is not only a passageway for the classical AF, but is also a convergence zone for several noncanonical association fibers. Among them, are the middle longitudinal fasciculus (MdLF), the so‐called fronto‐parietal (SLFIII) and temporo‐parietal segments of the AF (SLFtp), the vertical occipital fasciculus (VOF), and the second branch of the superior longitudinal fasciculus (SLFII). Disentangling these pathways using traditional user‐dependent tractography methods will likely fall victim to methodological idiosyncrasies that is further compounded by the disagreements in pathway terminations (Mesulam, Thompson, Weintraub, & Rogalski, 2015) as well as in their proposed segmentations (Glasser & Rilling, 2008; Makris et al., 2013).

Here, we implement a computational framework that integrates recent advances in connectomics (Gleichgerrcht, Fridriksson, Rorden, & Bonilha, 2017; Fridriksson et al., 2018; Yourganov, 2016) and regularized machine learning models (Lê Cao, Martin, Robert‐Granié, & Besse, 2009) to algorithmically isolate the connectional neuroanatomy of speech repetition among the broader white matter feature space. By applying regularization penalties on high‐density connectomes, elements of the feature space (i.e., connections) can be identified on the basis of their predictive value with a response variable (Hastie, Tibshirani, & Wainwright, 2015) rather than through conventional univariate association methods (Fridriksson et al., 2018), which might not generalize to independent datasets. Generally, aphasia studies have been limited in sample size and given that conduction aphasia is relatively rare among the aphasia taxonomies, this limitation has been especially pronounced when investigating its underlying biological mechanisms. To approach this problem, we measured repetition performance in a relatively large sample of subjects with stroke induced aphasia. By comprising a multitude of aphasia classifications, this cohort presents a unique opportunity to identify associations between white matter disconnection and speech repetition impairment, as each of the subtypes vary considerably with respect to their cerebrovascular lesions as well as in the extent to which repetition is disrupted. This association was established using a regularized latent projection‐based algorithm—sparse Partial Least Squares‐Regression (sPLS‐R)—in order to select a subset of white matter connections most predictive of repetition performance; sPLS aims to generate a parsimonious model by discarding non‐informative features when optimizing prediction error (Lê Cao et al., 2009). In contrast to conventional univariate methods, sPLS uses dimensionality reduction via multivariate latent projections which accommodates both high‐dimensionality and collinearity of the feature space (Rohart, Gautier, Singh, & Lê Cao, 2017). Multicollinearity is a pertinent issue in voxel‐based (VLSM) and connectome‐based (CLSM) lesion studies wherein elements of the feature space are highly interdependent, as lesions tend to impact adjoining voxels—likely inflating the type I error rates (Arnoux et al., 2018). Although PLS is a well‐established method in neuroimaging analysis (McIntosh, Bookstein, Haxby, & Grady, 1996), to our knowledge, it is regularized counterpart—used in high‐throughput “Omics” research from computational biology (Lê Cao, Boitard, & Besse, 2011)—has not yet been applied to study the *connectOmics* of speech and language (Sporns, 2013). The objective of the present study is to implement sPLS on the high‐dimensional connectome to first generate a parsimonious model of white matter features which will then, in turn, be used to generate predictions for speech repetition on an independent test set. By algorithmically ranking the most informative features based on their predictive value, we believe this approach will isolate the white‐matter correlates of the speech repetition circuit in a data‐driven, spatially unbiased manner.

## 2 METHODS

### 2.1 Participants, behavioral evaluations, and outcome measure

Data from seventy‐one participants (27 females, mean age = 60.37 ± 11.3, 7 with atypical handedness) with chronic aphasia (≥12 months post‐stroke) were analyzed here. Participants were recruited as part of a larger aphasia treatment study conducted at the University of South Carolina and Medical University of South Carolina. Only individuals with aphasia resulting from ischemic or hemorrhagic stroke to the left hemisphere were included. Participants with lacunar infarcts or with damage that involved the brainstem or cerebellum were excluded. All study procedures were approved by Institutional Review Boards at both institutions.

As part of this trial, individuals completed an extensive battery of cognitive‐linguistic testing and neuroimaging at baseline and at various post‐treatment time points (Kristinsson et al., 2021). Details about this trial can be found in Spell et al. (2020). All data included here were obtained at baseline, before initiating treatment. To test for repetition ability, the Philadelphia Repetition Test (Dell, Martin, & Schwartz, 2007) was used which evaluates repetition at a single‐word level. The PRT is a modification of the Philadelphia Naming Test (PNT) in which participants hear a pre‐recorded audio file and are asked to repeat exactly what they hear. In addition, the repetition subtest of the revised Western Aphasia Battery‐Revised (WAB‐R) was used to evaluate repetition at both the single‐word and phrase level (Kertesz, 2007). The scores for the PRT and WAB‐R were then normalized into percentages by dividing the respective columns by their maximum value. These measures were strongly correlated (*r* = .84), and were thus averaged to form a single composite score representing the participant's ability to perform repetition tasks at both the single word and phrasal levels. Lastly, subsequent analyses were performed on the composite repetition scores after correcting for the effects of age and months post‐stroke. Of the 71 participants enrolled in this study, the following seven aphasia subtypes were observed: Broca's (34 participants), anomic (15 participants), conduction (10 participants), Wernicke's (4 participants), global (4 participants), Transcortical‐Motor (1 participant), and no aphasia (3 participants). Mean aphasia severity (measured using the WAB Aphasia Quotient) was 58.31 (SD = 23.69, range 14.5–99.6). Additional participant details can be found in Table 1.

**TABLE 1:** List of relevant clinical and repetition measures assessed

Variable	Mean	*SD*	Range
WAB AQ	58.31	23.69	14.5–99.6
WAB repetition subscore	5.16	2.91	0.1–10
PRT score	105.21	59.64	0–175
Mean repetition score (%)	0.56	0.30	0.005–0.99
Months post‐stroke	53.76	57	12–241
Age at assessment	60.39	11.29	29–76

*Note*: List of relevant clinical and repetition measures assessed.

Abbreviations: WAB = Western Aphasia Battery; PNT = Philadelphia Repetition Test.

### 2.2 Image acquisition, lesion mapping, and preprocessing

Imaging was acquired on a Siemens Prisma 3 T scanner equipped with a 20‐element head/neck (16/4) coil at the University of South Carolina and Medical University of South Carolina. Images were generally acquired within 2 days of behavioral testing. This study used whole‐brain T1‐weighted, T2‐weighted, and diffusion echo planar imaging (EPI) images collected from each participant. Parameters were as follows:

- T1‐weighted image utilizing an MP‐RAGE sequence with 1 mm isotropic voxels, a 256 × 256 matrix size, a 9° flip angle, and a 192 slice sequence with repetition time = 2,250 ms, inversion time = 925 ms, echo time = 4.11 ms with parallel imaging (GRAPPA = 2, 80 reference lines).
- T2‐weighted image utilizing a sampling perfection with application optimized contrasts using a different flip angle evolution (3D‐SPACE) sequence. This 3D turbo spin echo (TSE) scan uses a repetition time of 3,200 ms, an echo time of 567 ms, variable flip angle, 256 × 256 matrix with 176 slices, 1 mm isotropic voxels, and parallel imaging (GRAPPA = 80 reference lines).
- Diffusion mono‐polar EPI scan that uses 43 volumes sampling 36 directions with *b* = 1,000 s/mm2 (with seven volumes *b* = 0), repetition time = 5,250 ms, echo time = 80 ms, 140 × 140 matrix, 90° flip angle, 80 contiguous slices, 1.5 mm isotropic voxels. This sequence was acquired twice, with phase encoding polarity reversed for the second series (anterior to posterior in the first series, posterior to anterior in the second series) in preparation for spatial undistortion with FSL Topup script.

The chronic post‐stroke lesions were manually drawn on the T2‐weighted image by a stroke neurologist (L.B.) or by a researcher with extensive experience with brain imaging in stroke populations; both were blinded to behavioral scores at time of lesion drawing. Using SPM12 and MATLAB scripts developed in‐house, the binary stroke lesion maps were spatially normalized to standard space through the following steps: (i) The T2 scan was co‐registered with the individual's T1 scan with the transforms used to resliced the lesion into native T1 space; (ii) the resliced lesion maps were smoothed with a 3 mm full‐width at half‐maximum Gaussian kernel to remove jagged edges associated with manual drawing; (iii) an enantiomorphic normalization (Nachev, Coulthard, Jäger, Kennard, & Husain, 2008) approach using SPM12's unified segmentation‐normalization (Ashburner & Friston, 2005) was applied to normalize the T1‐weighted images onto the standard space, using a chimeric T1‐weighted image where the area corresponding to the stroke lesion was replaced by the mirrored equivalent region in the intact (right) hemisphere; and (iv) the lesion mask was then binarized, and only voxels with a values of at least 0.5 were maintained in the final normalized lesion mask (Wilmskoetter et al., 2019).

### 2.3 Structural connectome analysis

Diffusion images were undistorted using TOPUP (Andersson, Skare, & Ashburner, 2003) and Eddy (Andersson & Sotiropoulos, 2016). Tractography was estimated using FSL's FMRIB's Diffusion Toolbox (FDT) probabilistic method (Behrens, Berg, Jbabdi, Rushworth, & Woolrich, 2007) with FDT's accelerated BEDPOST (Hernández et al., 2013) being used to assess default distributions of diffusion parameters at each voxel, and probabilistic tractography was performed using FDT's probtrackX (parameters: 5000 individual pathways drawn through the probability distributions on principal fiber direction, curvature threshold set at 0.2 (80**°**), 200 maximum steps, step length 0.5 mm, and distance correction). The waypoint mask was set as the white matter probabilistic map excluding the stroke lesion. The weighted connectivity between the regions *i* and *j* was defined as the number of probabilistic streamlines arriving at *j* region of interest when *i* was seeded, averaged with the number of probabilistic streamlines arriving at *i* region of interest when *j* was seeded. The connection weight was corrected based on the distance traveled by the streamlines connecting *i* and *j* (probtrackX's “distance correction”). The number of streamlines connecting each pair of regions of interest was further divided by the sum of the volumes of these regions of interest to compensate for the unequal size of gray matter regions of interest.

The structural connectome was defined with respect to the atlas of intrinsic connectivity of homotopic areas (AICHA), a fine‐grained parcellation containing 192 regions‐of‐interest (ROIs), of which 122 are gyral, 50 are sulcal, and 20 are gray nuclei (Joliot et al., 2015). Thus, a 192 × 192 symmetric connectivity matrix was generated per subject (36,864 total elements). The elements of this matrix represented the pairwise probabilistic streamline counts, normalized both by the distance between the ROIs and the volume of the two ROIs. The lower triangular of the matrix was extracted for each subject, vectorized, and concatenated into a 71 × 18,240 (subject by connection) matrix. This matrix was to be used as the feature space for subsequent analysis, where each column represented a particular pairwise connection between two AICHA ROIs and each row represented a particular subject. Lastly, to further eliminate near‐zero‐variance features or features with low connectivity values across the entire population, the matrix was thresholded to remove any connections with a mean normalized connectivity value of less than one, thereby reducing the feature space to a 71 × 2,363 matrix. Given the implications of the superior longitudinal fasciculus (SLF) in the classical literature, connections representing the three segments of the SLF (Forkel et al., 2014) were included regardless of the aforementioned threshold. These included any connections from inferior frontal and lateral temporal cortex, inferior frontal and supramarginal gyrus, and supramarginal gyrus to lateral temporal cortex. These SLF connections resulted in 228 additional connections, which finalized the feature space at 2,591 total connections for subsequent analysis.

### 2.4 Sparse partial least squares regression (sPLS‐R)

Partial Least Squares (PLS) is a multivariate latent projection‐based method in which a data matrix is integrated with a response vector via latent structures and, unlike traditional methods, is effective at modeling noisy, collinear datasets (described in Wold, Sjöström, & Eriksson, 2001). Whereas Principal components Analysis (PCA) projects a data matrix onto vectors corresponding to the direction of maximum variance (unsupervised), PLS projects the data matrix onto vectors which maximize covariance with the outcome measure, and can therefore be seen as the supervised counterpart to PCA (Kuhn & Johnson, 2013). The resulting PLS components may then be used to generate predictions through classical regression (called PLS‐Regression). More recently, regularization penalties have been incorporated into PLS projections to impose sparsity constraints during the dimensionality reduction, thereby enabling both model building and variable selection in a single step (Lê Cao et al., 2011). This approach, known as sparse PLS (sPLS), algorithmically performs feature selection during the model tuning procedure and results in a lower dimensional latent structure of predictive features. The appeal behind PLS in general, and sPLS in particular, is that it is not a so‐called “black box” algorithm and one is able to interpret either the weights of the loadings going into the principal PLS projections or the variable importance in the projection (VIP) coefficient (see next section) (Brereton & Lloyd, 2014). sPLS‐R was implemented using the “mixomics” package in R, dedicated to the multivariate analysis of biological “omics” datasets (Rohart et al., 2017). First, a 70/30 split was performed on the entire dataset such that 50 subjects (70% of the dataset) would be used to build a model whose predictions could then be tested on the remaining 21 subjects (30% of the dataset). The hyperparameters for sPLS are the number of components to retain (*H*) and the ℓ1‐regularization penalty (keepX), selected on the basis of optimizing prediction accuracy using mean absolute error). Here, we chose to fix the *H* at five while evaluating a list of different penalties with a maximum *keepX* of 100, tuned using repeated k‐fold cross validation (*k* = 5, repeats = 50).

### 2.5 Variable selection using the Bootstrap‐variable importance in projection (VIP) approach

In order to rank the retained features on the basis of their predictive value, the variable importance in projection (VIP) method was used (Chong & Jun, 2005; Farrés, Platikanov, Tsakovski, & Tauler, 2015). VIP is regarded as the impact of a given variable into the construction of the PLS components, weighted by the explained variance across the components. Features with a large VIP score, larger than one, have been shown to indicate relevance for explaining the outcome measure and this cutoff is widely used as a criterion for variable selection (Chong & Jun, 2005; Colombani et al., 2012; Farrés et al., 2015).

In order to establish distributions around the VIP estimates, the bootstrap‐VIP method (Gosselin, Rodrigue, & Duchesne, 2010) was implemented wherein the sPLS model tuning procedure was replicated 8,000 times using bootstrap resamples of the training set and the VIP scores for the principal PLS component at each iteration was recorded. If a variable is truly important in predicting the outcome measure, we may expect it to not only consistently survive the regularization penalty but also maintain a relatively strong VIP score across the different pseudo‐independent datasets (Afanador, Tran, & Buydens, 2013; Colombani et al., 2012). For this reason, the candidate pathways for subsequent analysis were identified using the aforementioned “greater than one” threshold on the mean of the bootstrap distribution (Chong & Jun, 2005; Farrés et al., 2015), while imposing that these features survived the regularization penalty on the majority of resampling iterations (selection stability frequency greater than 50%) (Lê Cao et al., 2011; Tillisch et al., 2017). Lastly, the resulting features were used to fit a PLS model on the training set and its predictions were then evaluated for statistical significance using their correlations with repetition scores from the unseen test set.

## 3 RESULTS

**FIGURE 1:**
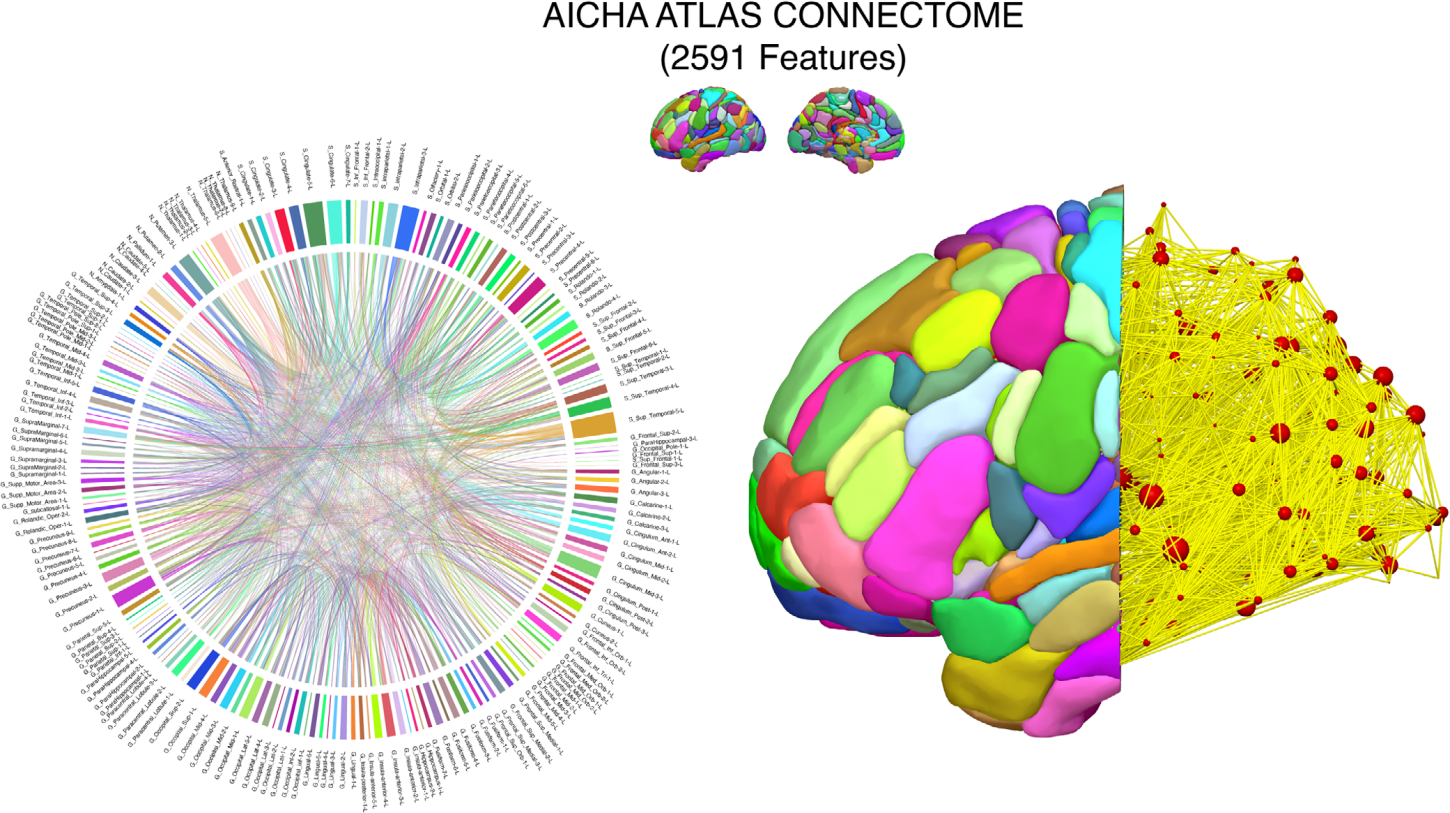
Connectomic feature space. A chord diagram representing the complete feature space of 2,591 white matter connections analyzed in this study. Each colored rectangular bar along the circumference of the circle represents a particular region of interest defined by the AICHA atlas and the interconnecting arches or lines represent the connections between region pairs generated with probabilistic tractography. This connectome was mapped for each of the 71 participants, resulting in a 71 × 2,591 data matrix

**FIGURE 2:**
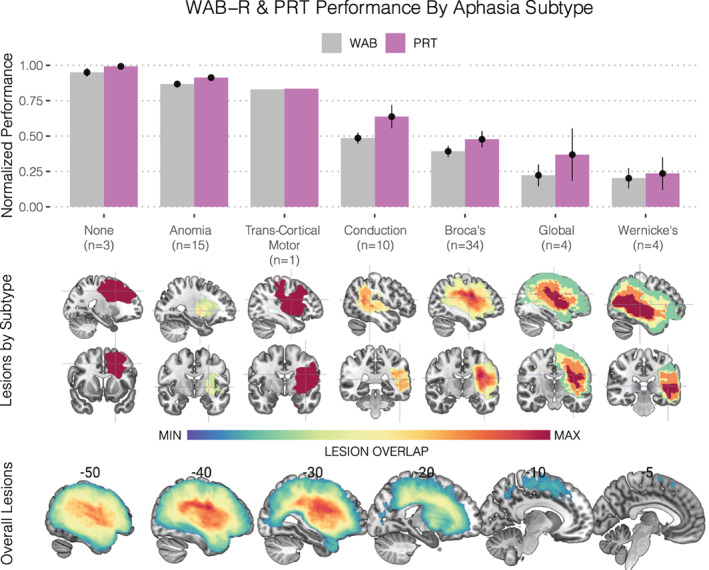
Repetition performance by aphasia subtype. (Upper) Barplots and respective standard errors of the normalized behavioral performances for the two speech repetition tasks administered: (1) the Western Aphasia Battery—Revised (WAB‐R) subtest for repetition (shown in gray) and (2) the Philadelphia Repetition Test (PRT) (shown in violet). These scores were normalized by dividing them by the maximum. Bars are grouped according to their respective aphasia taxonomies and sorted in descending order based on performance. Given the strong correlation between these scores (*r* = .84), performance was averaged prior to subsequent analyses. (Middle) Lesion overlap maps for each subtype are displayed beneath the *x*‐axis with their respective crosshairs being centered on the center of mass to enable visualization. (Lower) Overall lesion overlap maps across the entire cohort are displayed along with their respective sagittal slice coordinates in MNI space

The complete feature space of 2,591 connections is displayed as a chord diagram in Figure 1 using the *circlize* package in R (Gu, Gu, Eils, Schlesner, & Brors, 2014), whose arcs represent each of the distinct connections between brain regions defined by the AICHA atlas (Figure 1). The behavioral scores on the WAB‐R and PRT for all participants—shown in Figure 2—show the marked variability in behavioral performance within and between several of the aphasia classifications as well as the distribution of lesions within each subtype (Figure 2). This pattern of variability supports our approach to model a continuous outcome for repetition performance rather than differentiate aphasia classifications categorically. Nevertheless, the aphasia subtypes with most severe repetition deficits were observed in the Conduction, Broca's, Global, and Wernicke's aphasia subtypes; these groups presented with lesions impacting posterior perisylvian cortex to varying degrees. The lesions in the remaining groups were located relatively anterior to their repetition‐impaired counterparts, located predominantly in the frontal lobe and insular cortex (Figure 2, bottom). When adjusting for the effects of age and months post‐stroke, the former showed a positive but statistically weak association with repetition scores (*β* = 0.00086, *p* = .18), while the latter showed a relatively stronger but nonsignificant negative association (*β* = −0.006, *p* = .08) which together explained 7% of the total variance, *F*(2, 68) = 2.395 (*p* = .09887, multiple *R* ^2^ = 0.06579).

**FIGURE 3:**
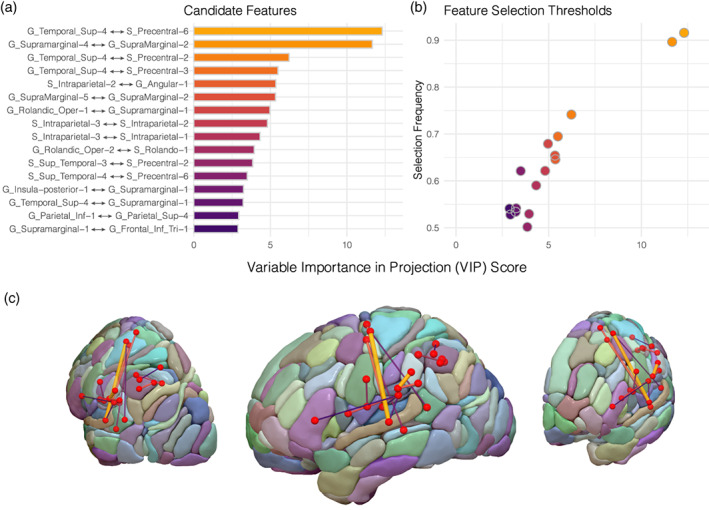
Bootstrap‐VIP variable selection results. (a) Variable importance in projection (VIP) scores of the candidate features averaged across the 8,000 bootstrap resampling iterations of the sparse partial least squares regression (sPLS‐R) algorithm applied on 70% of the dataset. (b) Features surviving the “greater than one” VIP (*x*‐axis) and greater than 50% selection frequency (*y*‐axis) thresholds are plotted, resulting in 16 features for subsequent analysis. (c) The 16 features are displayed as edges and are overlaid onto the AICHA atlas. Edges are scaled in diameter according to their respective bootstrap‐VIP scores

**FIGURE 4:**
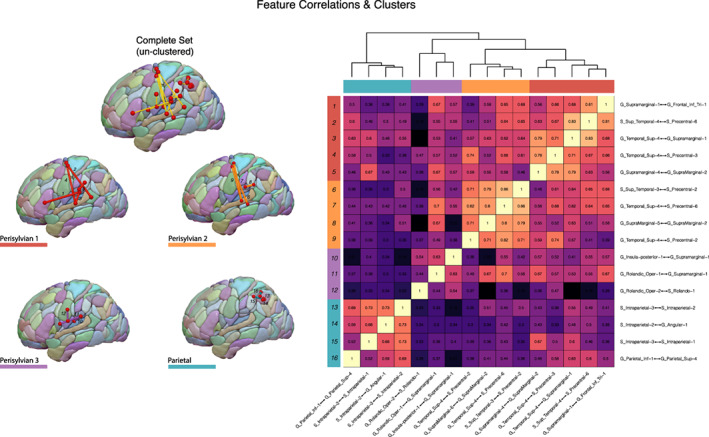
Feature correlations and clusters. (Right) A clustered correlation matrix heatmap of the 16 candidate features illustrating the positive correlations among the feature space. The features could be clustered into four distinct groups with shared connectivity patterns—as indicated by the cluster dendrogram—and these clusters are displayed as color‐coded edges on the AICHA atlas for visualization (Left). Each of the individual edges are labeled numerically, corresponding to the row labels of the correlation heatmap for identification

Visualizing the bootstrap‐VIP distributions from 8,000 resampling iterations applied on the training set (70% of the dataset), sixteen features (i.e., connections) were identified as candidates by surviving the greater than one mean VIP threshold and greater than 50% selection frequency threshold (Figure 3a,b). Of these sixteen candidate features selected, fifteen (94%) had terminations within parieto‐temporal nodes (Figure 3c); namely in supramarginal (38%), superior temporal (31%), and inferior parietal areas (25%). The remaining feature was a connection between ventral rolandic areas (G_Rolandic_Oper‐2 and S_Rolando‐1). The two features showing the strongest averaged bootstrap‐VIP score (VIP > 10) and selection frequency (Freq ≥ 90%) was a long‐range connection between the superior temporal gyrus and the dorsal precentral sulcus (G_Temporal_Sup⟷4S_Precentral‐6; VIP = 12.3, Freq = 92%) and a short‐range connection within the supramarginal gyrus (G_SupraMarginal‐4⟷G_SupraMarginal‐2; VIP = 11.66, Freq = 90%). The identified features were all positively correlated as shown by the symmetric connectivity matrix (Figure 4, right), and could be further characterized into highly correlated feature clusters using a hierarchical clustering algorithm. This unsupervised approach identified two predominant parietal and perisylvian clusters with similar connectivity patterns, with the latter being further sub grouped into three smaller clusters as shown by the clustering dendrogram (Figure 4, left). Perisylvian clusters 1 and 2 contained similar elements of fronto‐temporal and supramarginal connections, with the notable difference being that the former contained connections characteristic of the classic arcuate fasciculus as shown by the connections interconnecting pars triangularis of the inferior frontal gyrus with the superior temporal sulcus. Perisylvian cluster 3 contained predominantly rolandic connections while the parietal cluster contained short‐range connections constrained to the parietal lobule (Figure 4).

**FIGURE 5:**
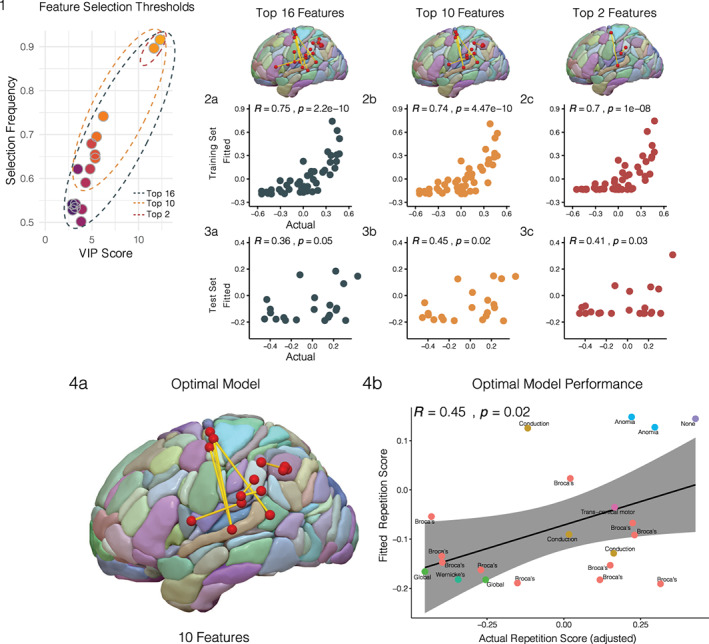
PLS model selection. (1) Three connectomic PLS models were evaluated by applying progressively higher feature selection thresholds on the 16 candidate features: a model containing the complete set of 16 features (dark gray), a model containing the top 10 features (orange), and a model containing the top two features (red). (2) The three models were trained on the training set (*n* = 50) (2a–c) and their performances were evaluated on the held out test set (*n* = 21) using correlations computed between fitted and actual repetition scores for each respective model (3a–c). (4) On the basis of its predictive performance on the test set (*R* = 0.45, *p* = .02), the connectomic PLS model containing the top 10 features was chosen as the optimal model (4a). The actual versus fitted scores of the optimal model are plotted with respect to the aphasia subtypes present in the test set (4b)

After identifying the candidate features using the VIP (VIP > 1) and selection frequency (Freq >50%) threshold, the predictive value of three different nested models were evaluated on the basis of their progressively higher threshold combinations (Figure 5, panel 1). The justification of this approach was to select the optimal model that produced predictions with statistically significant correlations on the unseen test set. The first model contained all 16 features identified by the feature selection protocol, the second contained the top 10 features, and the third contained the top 2 features (Figure 5). Each nested model was used to train a PLS model on the training set whose predictions were then evaluated on the held out test set (30% of the dataset). Of the three models evaluated on the training set, all made predictions that were significantly correlated with the adjusted repetition scores. Model 1 (16 features) showed the best performance on the training set (*R* = 0.75, *p* = 2.2e−10, one‐tailed) with models 2 (*R* = 0.74, *p* = 4.47e−10, one‐tailed) and 3 (*R* = 0.7, *p* = 1e−08, one‐tailed) similarly showing significant correlations on the training data (Figure 5, panel 2a–c). Of these three models, models 2 (*R* = 0.45, *p* = .02, one‐tailed) and 3 (*R* = 0.41, *p* = .03, one‐tailed) generalized their performance by achieving statistically significant correlations on the unseen test set. The features of the optimal model comprised four connections between the superior temporal lobe and dorsal precentral cortex, three connections with terminations in the supramarginal gyrus, and the remaining three were short‐range connections localized to the inferior parietal cortex posterior to the supramarginal features (Figure 5, panel 4a). A scatterplot between the optimal model fitted predictions and the actual (adjusted) repetition scores are displayed in Figure 5 (Figure 5, panel 4b), with each point color‐coded with respect to the aphasia subtype of each respective subject in the test set. The PLS loadings and variable importance in projection scores for the optimal model's latent projection are shown in descending order in Table 2. Four total features had VIP coefficients greater or equal to one, indicating their preferential significance when constructing the principal PLS projection. Three of which were long‐range connections between the superior temporal gyrus and the dorsal precentral cortex, but with the strongest coefficient (loading = 0.35, VIP = 1.1) combination being attributed to the short‐range supramarginal connection (G_SupraMarginal‐4⟷G_SupraMarginal‐2) (Table 2).

**TABLE 2:** PLS component coefficients and feature statistics

Structural connection	Loadings	VIP	Mean (*SD*)	Range
G_Supramarginal‐4↔G_SupraMarginal‐2	0.35	1.1	2.12 (3.28)	13.22
G_Temporal_Sup‐4↔S_Precentral‐6	0.34	1.09	0.01 (0.03)	0.15
G_Temporal_Sup‐4↔S_Precentral‐3	0.33	1.05	0.01 (0.03)	0.14
S_Intraparietal‐3↔S_Intraparietal‐2	0.32	1.02	18.89 (15.04)	73.28
G_Temporal_Sup‐4↔S_Precentral‐2	0.32	1	0.02 (0.04)	0.23
S_Sup_Temporal‐4↔S_Precentral‐6	0.31	0.96	0.02 (0.05)	0.26
G_Rolandic_Oper‐1↔G_Supramarginal‐1	0.3	0.96	1.12 (2.38)	9.04
G_SupraMarginal‐5↔G_SupraMarginal‐2	0.3	0.94	1.5 (3.03)	17.96
S_Intraparietal‐3↔S_Intraparietal‐1	0.3	0.94	9.01 (9.41)	38.73
S_Intraparietal‐2↔G_Angular‐1	0.29	0.9	5.35 (6.55)	28.08

*Note*: PLS component coefficients and feature statistics. Loadings and variable importance in projection (VIP) coefficients of the principal component resulting from the partial least squares (PLS) model fit on the optimal 10 features across the entire dataset (*n* = 71). Means, standard deviations, and ranges are also reported for each individual feature.

**FIGURE 6:**
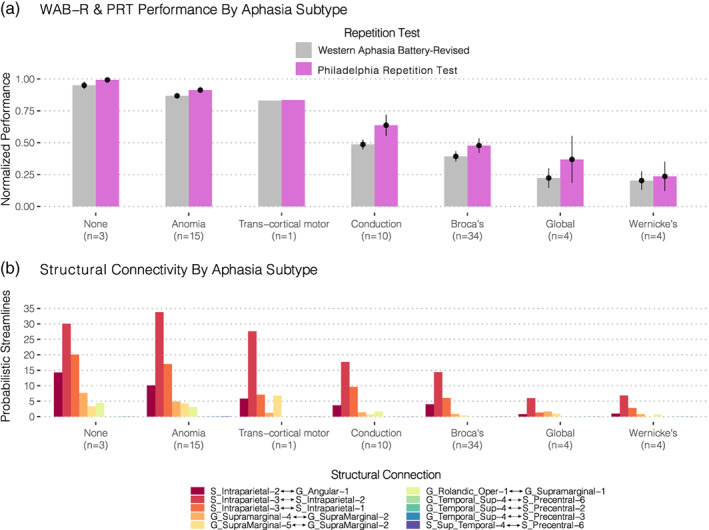
Connectome‐Symptom Mapping. Using the same descending order shown and described in Figure 2, barplots of the normalized probabilistic streamline counts are shown across the entire group (*N* = 71) for each of the optimal 10 features identified. Both the behavioral patterns and structural connectivity measures show similar descending patterns across the aphasia taxonomies

The connectivity values of the 10 identified parieto‐temporal white matter features were then inspected across the aphasia taxonomies in the entire group of 71 subjects (Figure 6b). Sorted by the same descending order of aphasia taxonomies based on repetition performance (Figure 2), the pattern of descending connectivity values shows a markedly similar descending pattern. Subtypes with better performance on the composite repetition test score showed greater probabilistic streamlines in the identified parieto‐temporal connections, indicating that the associated brain lesions spared these pathways. In contrast, subtypes with greater repetition impairment showed lower streamline counts in the identified pathways likely due to disruption caused by the respective lesion.

## 4 DISCUSSION

Using a high‐dimensional connectomics approach combined with algorithmic feature selection, the present study isolated the white matter substrates for speech repetition among the broader set of cerebral white matter. By obviating the need to restrict analysis to a priori selected pathways, we show how modern machine learning algorithms could leverage connectomics data to generate novel hypotheses for uncovering brain‐behavior relationships when studying the neurobiology of language. It is worth emphasizing that most—if not all—studies on the white matter correlates of speech repetition have focused exclusively on the classical arcuate fasciculus (AF) given its implication from classical neurobiological models (Berthier et al., 2012; Forkel et al., 2020; Yeatman et al., 2011). The connectomic approach implemented here identified short‐ and long‐range connections emanating from the superior temporal cortex and parieto‐temporal junction as essential for repetition function (Figure 3). Of the connections identified, an optimal subset of 10 white matter features were capable of making significantly accurate predictions on the unseen test set—thereby attesting to the generalizability of our findings (Figure 5, panel 4). Fifty percent of the optimal features were short‐range connections in the inferior parietal lobe, with preferential contributions from short supramarginal white matter closely delineating cortical area Spt (Table 2), and with the remaining set highlighting longer range connections terminating in ventral and, most notably, in dorsal premotor cortex (Figure 5, panel 4a). Contrary to the Wernicke‐Geschwind theoretical framework, our analysis strategy offers a rather nuanced architecture of the dorsal stream's white matter circuitry—apart from the role of the classical arcuate fasciculus—that is more in line with findings from the functional imaging literature (Buchsbaum et al., 2011). Our study supports the notion that repetition is carried out by cortico‐subcortical circuits within the parieto‐temporal cortex along with fronto‐parieto‐temporal pathways with frontal terminations not limited to Broca's area. Importantly, all identified pathways shared an anatomical feature in common—their posterior terminations in parietal or temporal cortex, further cementing this region as critical for the sensorimotor transformations required to repeat aurally presented linguistic stimuli.

Consistent with modern imaging studies, our results underscore the importance of the parieto‐temporal cortex for the repetition of speech (Buchsbaum et al., 2011; Fridriksson et al., 2010; Hickok & Poeppel, 2007; Yourganov, Fridriksson, Rorden, Gleichgerrcht, & Bonilha, 2016). Lukic et al. (2019) have recently shown a relationship between the *cortical* thickness in parieto‐temporal cortex and repetition performance—a finding that has since been replicated by Forkel and colleagues (Forkel et al., 2020; Lukic et al., 2019). Similarly, Rogalsky and colleagues—using a VLSM approach—localized repetition impairments to focal brain damage in the same vicinity (Rogalsky et al., 2015). Combined with these studies and others (Buchsbaum et al., 2011; Hickok & Poeppel, 2007; Isenberg, Vaden Jr, Saberi, Muftuler, & Hickok, 2012), the present work highlights the significance of parieto‐temporal cortical area Spt (Hickok et al., 2003) and of its subjacent subcortical connections for auditory‐motor integration (Figure 3c). This claim was justified by the feature selection procedure, as nearly 70% of the candidate features had terminations in either superior temporal or supramarginal nodes (Figure 3). Furthermore, when fitting the PLS model using the optimal 10 features across the entire dataset (i.e., on the combined training and test set), the feature with the strongest VIP and loading coefficient was a short‐range connection within the supramarginal gyrus (G_Supramarginal‐4↔G_SupraMarginal‐2) which precisely overlaps with area Spt (Figure 5, Table 2) (Isenberg et al., 2012).

One of the distinct feature clusters sharing strongly correlated connectivity patterns was localized to the inferior parietal lobule (Figure 4, left), with connections spanning the intraparietal sulcus and angular gyrus. Indeed, several such connections of the intraparietal sulcus persisted when selecting the predictive model that generalized to the unseen test set (Figures 5 and 6). Interestingly, Geschwind had originally speculated that association cortex of the angular gyrus was likely involved in Wernicke's aphasia—later called Geschwind's territory (Catani & Mesulam, 2008)—and among the aphasia subtypes enrolled in this study, Wernicke's aphasics showed the poorest performing repetition scores (Figure 2). This group concomitantly showed the greatest disruption in the parieto‐temporal pathways; which included several short‐range connections in this territory (Figures 5 and 6). Similarly, Buchsbaum and colleagues have implicated the intraparietal sulcus (IPS) in sensorimotor integration by demonstrating fMRI activations of this area during both encoding and rehearsal phases of a verbal working memory task requiring participants to covertly rehearse aurally presented stimuli for subsequent recall (Buchsbaum et al., 2011). Their analysis showed relatively bilateral IPS fMRI activations when compared to the lateralized activations observed in area Spt. This might explain why the supramarginal features identified here showed relatively stronger PLS coefficients when fitting the optimal model (Figure 5) on the entire dataset (Table 2), such that the dorsal stream circuit appears to rely more on left‐lateralized parieto‐temporal elements as opposed to the less lateralized parietal areas.

In addition to the short‐range features identified in the parietal and temporal cortex, the present work stresses the importance of several long‐range connections originating from these areas and terminating in frontal premotor cortex (Figures 3 and 4). Notably, features interconnecting the essential posterior nodes of the superior temporal and supramarginal regions appeared to connect to ventral premotor as well as dorsal premotor areas of the frontal lobe (Figure 5, panel 4a). This finding points to a role of the superior longitudinal fasciculus, particularly of its sub‐branches (i.e., AF and SLFIII), as being involved in the dorsal repetition circuit. Perisylvian cluster 1 (Figure 4, left) contained connections between superior temporal and pars triangularis of the inferior frontal gyrus which precisely mirrors the trajectory of the arcuate fasciculus (Catani, Jones, & Ffytche, 2005). Perisylvian cluster 3 contained rolandic white matter interconnecting the supramarginal gyrus with the ventral premotor cortex (vPMC), which similarly approximates the third branch of the SLF (SLFIII) (Forkel et al., 2014). Interestingly, when the sixteen features were refined to retain the subset most predictive of performance in the test set, the SLFIII feature (G_Rolandic_Oper‐1↔G_Supramarginal‐1) was retained but the arcuate counterparts were not (Figure 5, panel 4a). This attributes a relatively stronger role for frontal pathways with posterior terminations directed to the supramarginal gyrus, for reasons discussed in the preceding paragraph. The SLFIII is therefore an essential neuroanatomical substrate for the dorsal language stream likely due to its role in enabling the conversion of auditory input stored in verbal working memory areas of the SMG into phonological and articulatory forms within the ventral premotor cortex (Duffau, 2015 for a review).

Another set of features highlighted by the present work are the contributions of a potentially novel set of fronto‐temporal connections terminating in the dorsal premotor cortex (Figures 3, 4, 5, 6). Across the feature selection iterations subjected to the bootstrap resampling procedure (Figure 3a), the feature with the strongest projection coefficient and selection frequency was a connection between the superior temporal gyrus and the dorsal precentral sulcus (G_Temporal_Sup‐4↔S_Precentral‐6). Moreover, three out of the top five candidate features terminated in this dorsal precentral region (Figure 3a) and among the ten features selected in the optimal predictive model, 40 % showed similar connectivity partners (Figure 5, panel a). Although the connectivity values of this pathway were relatively sparse in comparison to the other features identified (Figure 6), this connection set appears to heavily augment predictive power when considered in combination with adjacent short‐range connections. Indeed, of the three nested models evaluated on the test set, a model containing just two features (Figure 5, model 3) produced significantly correlated predictions with the test set data. This two‐feature model consisted of a short supramarginal connection and a long dorsal premotor connection.

To our knowledge, this is the first large‐scale study to implicate a structural connection to dorsal premotor cortex as being involved in repetition of speech. Classical neurobiological models of language make no mention of an area yet contemporary models appear to suggest a role for a dorsal premotor area in laryngeal motor control (Dichter, Breshears, Leonard, & Chang, 2018; Hickok, 2017). The dual stream theoretical model proposed by Hickok and Poeppel (2007) noted a close coupling between sensory‐motor area Spt and a dorsal premotor area involved in articulation (Hickok & Poeppel, 2007). Recently, functional imaging studies have localized an area in close proximity to this region—area 55b—which appears to activate during language tasks (Glasser et al., 2016). Similarly, recent direct electrical stimulation studies have shown that stimulation of this dorsal premotor region causes speech disturbances intraoperatively (Hazem et al., 2021; Rech et al., 2019) and this area is both functionally and structurally connected to the superior temporal cortex (Barbeau, Descoteaux, & Petrides, 2020; Rech et al., 2019). Moreover, neurosurgical evidence suggests that tumor resection within the dorsal premotor cortex can produce long‐term speech production deficits characterized by apraxia and impairments with repetition (Chang et al., 2020). These authors also demonstrated white matter connections to exist between this dorsal frontal area and the temporal lobe via the superior longitudinal fasciculus. Indeed, a recent (anatomical study) from Barbeau and colleagues have published evidence that a dorsal branch of the arcuate fasciculus exists in humans, which terminates in the posterior dorsolateral frontal region anteriorly and the superior temporal cortex posteriorly (Barbeau et al., 2020)—precisely the connectivity pattern identified in the present behavior‐connectomic analysis. Thus, we provide novel evidence of a structure–function relationship between this pathway and a prominent language function.

The association between conduction aphasia and apraxia has been shown particularly in the presence of supra‐sylvian lesions located deep to the inferior parietal lobe (IPL); specifically near the supramarginal gyrus (Basilakos, Rorden, Bonilha, Moser, & Fridriksson, 2015; Benson et al., 1973; Geschwind, 1965; Mendez & Benson, 1985; Poncet, Habib, & Robillard, 1987). This association may potentially be mediated by connectivity between area Spt and its communication with this dorsal premotor speech area via the 55b‐Spt pathway critical for laryngeal motor control. These data suggest that lesions to the IPL may disrupt communication between parieto‐temporal sensory‐motor cortex and dorsal premotor areas critical for buccofacial (Benson et al., 1973) and laryngeal motor control (Hickok, 2017), as these areas are functionally (Glasser et al., 2016; Rech et al., 2019) and structurally (Chang et al., 2020; Rech et al., 2019) interconnected. Among the 16 features identified in this study, the dorsal premotor‐superior temporal pathways showed the strongest correlations with the supramarginal connections (Figure 4) to the extent that they were clustered together (Perisylvian clusters 1 and 2), further suggesting that these connections might originate from posterior aspects of the superior temporal gyrus. Contrary to claims made by classical theoretical models, the posterior STG (i.e., anatomical Wernicke's area) is increasingly being recognized for its role in activating phonological representations rather than in the comprehension of speech (see Binder, 2017 for a review).

Considering the present work implicates short‐ and long‐range connections outside of the classical arcuate pathway, what is the functional role of the AF in the dorsal stream? The recent literature suggests that the AF might instead be essential for syntactic processing (den Ouden et al., 2019; Meyer, Cunitz, Obleser, & Friederici, 2014; Wilson et al., 2011) rather than for the sensorimotor functions needed for the repetition of speech. Considering that it connects posterior syntactic comprehension sites in the posterior middle temporal gyrus with anterior syntactic production sites in the posterior frontal cortex (i.e., in Broca's area) (Matchin & Hickok, 2020), this characterization appears more likely than it playing the major role in phonological aspects of speech. If the AF were indeed involved in repetition, we would expect to find that damage to its anterior termination in Broca's area would emerge as a strong cortical correlate in VLSM studies—a finding not supported by recent work (Rogalsky et al., 2015). Parietotemporal regions superior to Wernicke's area increasingly appear critical for auditory‐verbal working memory storage and rehearsal processes (Lukic et al., 2019; Rogalsky et al., 2015) in order to maintain complex stimuli online while simultaneously integrating with articulatory‐phonological areas that are coupled with laryngeal motor cortex via the Spt‐55b and Spt‐vPMC pathways identified here. Taken together, the literature and the present work support the notion that the posterior STG (Basilakos, Smith, Fillmore, Fridriksson, & Fedorenko, 2018) and its underlying connections, are involved in the sensory guidance of speech whose disruption drastically impairs both production and repetition (Figure 6).

## 5 CONCLUSION

The purpose of the present study was to isolate pathways critical for speech repetition using a data‐driven feature selection algorithm applied on the structural connectome. By evaluating this relationship in stroke patients presenting with varying lesions and varying degrees of impairment, this connectome‐based lesion symptom mapping approach successfully highlighted a focal set of superficial parieto‐temporal connections as being essential for the prediction of repetition performance. This finding corroborates classical and contemporary models by indicating that the repetition impairments observed in conduction aphasia might rather be characterized as a parietotemporal disconnection syndrome impacting cortical area Spt and associated frontal circuits as opposed to being explained as purely a disconnection of the classical arcuate fasciculus. The study also identified an additional important circuit involving superior temporal connectivity to a dorsal premotor site that calls for further investigation in the future. In conclusion, machine learning analyses frameworks offer the unique capacity to assess clinico‐anatomical correlations in a theoretically unbiased manner, leading to novel insights into the neurobiology of language without having to reduce the complexity of the underlying anatomical feature space that is often characteristic of high‐dimensional neuroimaging studies.

## Data Availability

The data used in this study are available to researchers upon qualified request to the corresponding author.
